# The Association between Oral Health-Related Quality of Life, Loneliness, Perceived and Objective Social Isolation—Results of a Nationally Representative Survey

**DOI:** 10.3390/ijerph182412886

**Published:** 2021-12-07

**Authors:** André Hajek, Hans-Helmut König

**Affiliations:** Hamburg Center for Health Economics, Department of Health Economics and Health Services Research, University Medical Center Hamburg-Eppendorf, 20246 Hamburg, Germany; h.koenig@uke.de

**Keywords:** oral health, oral health-related quality of life, loneliness, social isolation, social exclusion

## Abstract

The aim was to examine the association between oral health-related quality of life and loneliness and perceived as well as objective social isolation. Data were used from a nationally representative survey with n = 3075 (late Summer 2021). The established Oral Health Impact Profile (OHIP-G5) was used to quantify oral health-related quality of life. Moreover, established tools were used to quantify the outcome measures (De Jong Gierveld loneliness scale, Bude/Lantermann scale and Lubben Social Network Scale). It was adjusted for several covariates in regression analysis. Linear regressions showed that low oral health-related quality of life is associated with higher loneliness (B = 0.03, *p* < 0.001), higher perceived social isolation (B = 0.06, *p* < 0.001) and higher objective social isolation (B = 0.07, *p* < 0.05). Further regressions were performed (e.g., stratified by denture usage). Our study stressed the importance of low oral health-related quality of life for loneliness and social isolation (both perceived and objective). This knowledge is important to address individuals at risk. Future studies should clarify the underlying mechanisms.

## 1. Introduction

Loneliness, perceived social isolation and objective social isolation are gaining increasing awareness in the focus of research and the public—particularly in times of the COVID-19 pandemic, where social distancing is a main challenge. These aforementioned social needs are associated with subsequent morbidity and mortality [1,2]—which underlines the importance of these factors.

Various studies have identified the determinants of loneliness, perceived social isolation and objective social isolation [1,2]. Such determinants include sociodemographic factors, lifestyle-related, psychological and health-related factors [3,4,5,6].

However, thus far, there is limited knowledge regarding the association between oral health-related quality of life and these social needs.

Most of the existing studies in this area focused on the association between objective oral health (e.g., decayed teeth or dental caries) [7,8,9,10,11,12,13] rather than (perceived) oral health-related quality of life [14,15] and social needs (in terms of loneliness, perceived social isolation and objective social isolation). Most of the studies found an association between oral health and loneliness. However, existing studies often used data from small samples in quite specific regions and specific age groups (e.g., children or individuals in late life). In sum, there is a lack of studies analyzing the association between oral health-related quality of life and loneliness, perceived social isolation and objective social isolation based on data from the general adult population using nationally representative samples. Our aim was, therefore, to examine the association between oral health-related quality of life and loneliness, perceived social isolation as well as objective social isolation. This in turn is important to identify individuals at risk for high loneliness, perceived social isolation and objective social isolation.

The three social needs refer to different concepts. The outcomes are somewhat correlated (please see the Results section), but did not measure the same phenomenon. While loneliness refers to the discrepancy between actual and desired social relations [16], perceived social isolation refers to emotions of being left out from society [17] and objective social isolation refers to infrequent social contacts or rare social activities [18]. For further details, please see [19].

## 2. Materials and Methods

### 2.1. Sample

For our study, data were derived from a nationally representative online survey covering individuals aged 18 to 70 years. All participants lived in Germany. The sample size equaled 3075 individuals. It was performed from late August (24th) to early September (3rd). A well-established market research company recruited the participants. An online sample was used in a way that it matches the distribution of gender, age and federal state in the German population [20].

### 2.2. Outcomes

Loneliness was quantified using the De Jong Gierveld Loneliness scale (six-item version) [21]. Three items were recoded. The score was calculated by averaging the items. The score ranges from 1 to 4, with higher values indicating higher loneliness. In our study, Cronbach’s alpha was 0.78. It has been shown that this widely used tool (e.g., [22,23,24]) has good to very good psychometric characteristics [21,25].

The Bude and Lantermann tool (with four items) was used to measure perceived social isolation [17]. All items were recoded. A score was computed by averaging the items (from 1 to 4, higher values correspond to higher perceived social isolation). In our study, Cronbach’s alpha was 0.92.

Moreover, the six-item version of the Lubben Social Network Scale [26] was used to quantify objective social isolation. It should be highlighted that all items were recoded prior to computing a sum score for reasons of consistency. Consequently, the resulting sum score ranges from 0 to 30, with higher values reflecting higher objective social isolation. In our study, Cronbach’s alpha was 0.87. This scale has favorable psychometric characteristics [26].

### 2.3. Independent Variables

The established Oral Health Impact Profile (OHIP-G5) was used to assess oral health-related quality of life. It covers the four dimensions: (i) oral function, (ii) orofacial pain, (iii) appearance and (iv) psychosocial impact. It has good psychometric properties [27]. The OHIP-G5 ranges from 0 to 20, with higher scores indicating lower oral health-related quality of life. In our study, Cronbach’s alpha was 0.85. A further item refers to denture usage (options: 1. without dentures, 2. with removable dentures; 3. with complete dentures). Associations between oral health-related quality of life and the outcome measures can, thus, be stratified by denture usage, since it already proved to be a key predictor in the longer OHIP-G-versions [28,29].

It was adjusted for covariates as follows: sex, age, presence of children under 18 years in the same household (no; yes), marital status (married, living together with spouse; married, not living together with spouse; single; widowed; divorced), highest educational degree (upper secondary school; qualification for applied upper secondary school; polytechnic Secondary School; intermediate Secondary School; Lower Secondary School; currently in school training/education; without school-leaving qualification) and employment status (full-time employed; retired; other). Moreover, it was adjusted for smoking status (yes, daily; yes, sometimes; no, not anymore; never smoker), alcohol intake (daily; several times per week; once a week; 1–3 times per month; less often; never) and sports activities (no sports activity; less than one hour a week; regularly, 1–2 h a week; regularly, 2–4 h a week; regularly, more than 4 h a week). Furthermore, vaccination against COVID-19 (no; yes), self-rated health (from 1 = very bad to 5 = very good) and chronic diseases (absence of chronic diseases; presence of at least one chronic disease) were used as covariates.

### 2.4. Statistical Analysis

First, sample characteristics are shown stratified by oral health-related quality of life (dichotomized, first category: score of zero; second category: scores of one or higher). Thereafter, bivariate correlations are displayed (additionally stratified by denture usage). Subsequently, multiple linear regressions were performed with oral health-related quality of life as key independent variable (first regression: with loneliness as outcome measure; second regression: with perceived social isolation as outcome measure; third regression: with objective social isolation as outcome measure). Further regressions were calculated and stratified by denture usage. The level of significance was set at *p* < 0.05. Stata 16.1 (Stata Corp., College Station, TX, USA) was used for performing statistical analyses.

## 3. Results

### 3.1. Sample Characteristics

In our total sample, the mean age equaled 44.5 years, SD: 14.8 years. It ranged from 18 years to 70 years and 51.1% were female.

The average OHIP-G5 score was 2.2 (SD: 3.3), ranging from 0 to 20. Stratified by denture usage, the average OHIP-G5 score was 1.9 (SD: 3.0, 0 to 20) among individuals without dentures, 4.0 (SD: 4.5, 0 to 17) among individuals with removable dentures and 3.3 (SD: 3.8, 0 to 16) among individuals with complete dentures.

Stratified by oral health-related quality of life, sample characteristics are further displayed in Table 1. Dichotomized oral health-related quality of life was associated with most of the other variables (except for age, presence of children under 18 years in the same household, alcohol intake and vaccination against COVID-19).

### 3.2. Bivariate Analysis

It is worth noting that all outcomes are somewhat correlated (loneliness and perceived social isolation: r = 0.56, *p* < 0.001; loneliness and objective social isolation: r = 0.44, *p* < 0.001; perceived social isolation and objective social isolation: r = 0.27, *p* < 0.001). Additionally, oral health-related quality of life was associated with loneliness (r = 0.23, *p* < 0.001), perceived social isolation (r = 0.30, *p* < 0.001) and objective social isolation (r = 0.08, *p* < 0.001). Please also see Table 2.

Stratified by denture usage (1. without dentures; 2. with removable dentures; 3. with complete dentures), pairwise correlations were as follows: oral health-related quality of life was associated with loneliness (1: r = 0.24, *p* < 0.001; 2: r = 0.20, *p* < 0.001; 3: r = 0.28, *p* < 0.01), perceived social isolation (1: r = 0.29, *p* < 0.001; 2: r = 0.40, *p* < 0.001; 3: r = 0.38, *p* < 0.001) and objective social isolation (1: r = 0.08, *p* < 0.001; 2: r = −0.03, *p* = 0.58; 3: r = 0.08, *p* = 0.42). Thus, among individuals with complete dentures, oral health-related quality of life was only associated with loneliness. Please also see Table 3.

### 3.3. Regression Analysis

Findings of multiple linear regressions for the total sample are shown in Table 4.

Regressions showed that low oral health-related quality of life is associated with higher loneliness (B = 0.03, *p* < 0.001), higher perceived social isolation (B = 0.06, *p* < 0.001) and higher objective social isolation (B = 0.07, *p* < 0.05).

When the OHIP-G5 was dichotomized (first category: score of zero; second category: scores of one or higher), scores of one or higher were associated with higher loneliness (B = 0.20, *p* < 0.001), higher perceived social isolation (B = 0.28, *p* < 0.001) and higher objective social isolation (B = 0.46, *p* < 0.05) (please see Supplementary Table S1).

Stratified by denture usage, multiple linear regressions are shown in Table 5.

Among individuals without dentures, oral health-related quality of life was associated with higher loneliness (B = 0.04, *p* < 0.001), higher perceived social isolation (B = 0.06, *p* < 0.001) and higher objective social isolation (B = 0.08, *p* < 0.05).

Among individuals with removable dentures, oral health-related quality of life was associated with higher perceived social isolation (B = 0.05, *p* < 0.001), but not with loneliness and objective social isolation.

Among individuals with complete dentures, oral health-related quality of life was associated with higher loneliness (B = 0.04, *p* < 0.05) and higher perceived social isolation (B = 0.06, *p* < 0.01), but not with objective social isolation.

However, when examining the moderating effect of denture usage on the association between oral health-related quality of life and the outcomes (by including respective interaction terms), nearly all interaction terms did not achieve statistical significance (except for the interaction term between ‘with removable dentures’ and oral-health related quality of life, with loneliness as the outcome measure: B = −0.02, *p* = 0.02) (please see Supplementary Table S2).

## 4. Discussion

### 4.1. Main Findings

Based on data from a large, nationally representative sample, the aim of our study was to clarify the association between oral health-related quality of life and loneliness, perceived social isolation as well as objective social isolation. Pairwise correlations between oral health-related quality of life and all outcomes were small to medium (from 0.1 to 0.3). Regressions showed that low oral health-related quality of life is associated with higher loneliness, higher perceived social isolation and higher objective social isolation. Furthermore, associations were shown stratified by denture usage. Our study extends previous knowledge regarding the association between oral health-related quality of life and social needs mainly based on small samples in specific regions (and among children or individuals in late life).

### 4.2. Previous Research and Possible Explanations

Our study demonstrated an association between oral health-related quality of life and loneliness, perceived social isolation as well as objective social isolation.

With regard to the association between oral health-related quality of life and loneliness, our study is mainly in accordance with previous studies (typically based on small samples), showing an association between objective oral health [9,12] as well as oral-health related quality of life and loneliness [14].

While two studies exclusively focusing on children and adolescents did not find an association between objective oral health or oral health-related quality of life and objective social isolation [7,15], two other studies found an association between lower objective oral health and higher objective social isolation among older adults [8,11]. Furthermore, one study showed an association between lower objective oral health and higher objective social isolation among children [13]. Our study adds to this current knowledge based on samples from children or individuals in late life. Furthermore, we added knowledge regarding the association between oral health-related quality of life and perceived social isolation.

Several mechanisms may explain the association between oral health-related quality of life and the outcomes used in our study. For example, low oral health is associated with low mental health [30]. This in turn is associated with higher loneliness as well as higher social isolation [31,32]. Moreover, studies have demonstrated that oral health is associated with the risk of homebound status, which in turn can lead to feelings of loneliness or isolation [33]. Similarly, low oral health-related quality of life may increase factors such as shame, embarrassment and perceived stigma of being poor or may contribute to low self-esteem [34,35]—which in turn may lead to loneliness and isolation [36]. Additionally, factors such as social status, social class or mental health may be important for the association between oral health-related quality of life and the outcomes used in our study.

Our study also showed regressions stratified by denture usage. However, the sample size differed markedly between the groups. Thus, non-significant associations (particularly in the second and third group) may be particularly explained by a lack of statistical power.

### 4.3. Strengths and Limitations

For our study, data were used from a nationally-representative sample. Furthermore, as the first study in this area, we concentrated on the association between oral health-related quality of life and (i) loneliness, (ii) perceived social isolation and (iii) objective social isolation to get a more comprehensive picture. Widely used and well-established tools were used to quantify oral health-related quality of life and the social needs. It was adjusted for several potential confounders in regression analysis. Due to the cross-sectional design, it is difficult to draw causal conclusions. It also appears to be plausible that oral health-related quality of life contributes to loneliness or social isolation. Therefore, future longitudinal studies are needed in this area.

## 5. Conclusions

Our study showed the relevance of low oral health-related quality of life for loneliness and perceived social isolation as well as objective social isolation. Future studies should elucidate the underlying mechanisms. Moreover, future research is required to clarify the association between oral health-related quality of life and loneliness, perceived social isolation as well as objective social isolation in other countries.

## Figures and Tables

**Table 1:** Sample characteristics stratified by oral health-related quality of life (first category: score of zero; second category: scores of one or higher).

Variables		Oral Health-Related Quality of Life: Scores of Zero (N = 1537)	Oral Health-Related Quality of Life: Scores of One or Higher (N = 1538)	
				*p*-value
Sex: n (%)				0.07
	Men	782 (52.1)	720 (47.9)	
	Women	754 (48.0)	816 (52.0)	
	Diverse	1 (33.3)	2 (66.7)	
Age: mean (standard deviation)		44.3 (14.6)	44.8 (15.0)	0.41
Marital status: n (%)				0.03
	Single/Divorced/Widowed/Married, not living together with spouse	627 (47.8)	686 (52.2)	
	Married, living together with spouse	910 (51.6)	852 (48.4)	
Highest educational degree: n (%)				0.01
	upper secondary school	706 (53.2)	620 (46.8)	
	qualification for applied upper secondary school	161 (49.1)	167 (50.9)	
	polytechnic Secondary School	78 (46.4)	90 (53.6)	
	intermediate Secondary School	431 (48.5)	457 (51.5)	
	Lower Secondary School	157 (45.2)	190 (54.8)	
	currently in school training/education	1 (11.1)	8 (88.9)	
	without school-leaving qualification	3 (33.3)	6 (66.7)	
Children under 18 years in own household: n (%)				0.67
	No	1108 (50.2)	1098 (49.8)	
	Yes	429 (49.4)	440 (50.6)	
Migration: n (%)				
	No migration background	1395 (51.2)	1329 (48.8)	
	Migration background	142 (40.5)	209 (59.5)	
Employment status: n (%)				<0.001
	Full-time employed	797 (54.7)	661 (45.3)	
	Retired	210 (42.1)	289 (57.9)	
	Other	530 (47.4)	588 (52.6)	
Smoking: n (%)				<.001
	Yes, daily	328 (45.8)	388 (54.2)	
	Yes, sometimes	100 (39.8)	151 (60.2)	
	No, not anymore	402 (47.7)	441 (52.3)	
	Never smoker	707 (55.9)	558 (44.1)	
Sports activities: n (%)				<0.01
	No sports activity	409 (49.0)	425 (51.0)	
	Less than one hour a week	283 (45.0)	346 (55.0)	
	Regularly, 1–2 h a week	356 (49.9)	358 (50.1)	
	Regularly, 2–4 h a week	244 (51.6)	229 (48.4)	
	Regularly, more than 4 h a week	245 (57.6)	180 (42.4)	
Alcohol intake: n (%)				0.18
	Daily	84 (45.2)	102 (54.8)	
	Several times per week	272 (48.2)	292 (51.8)	
	Once a week	271 (54.7)	224 (45.3)	
	1–3 times per month	271 (50.9)	261 (49.1)	
	Less often	348 (48.7)	367 (51.3)	
	Never	291 (49.9)	292 (50.1)	
Vaccinated against COVID-19: n (%)				0.96
	No	297 (50.1)	296 (49.9)	
	Yes	1240 (50.0)	1242 (50.0)	
Chronic diseases: n (%)				<0.001
	Absence of chronic diseases	991 (56.1)	774 (43.9)	
	Presence of at least one chronic disease	546 (41.7)	764 (58.3)	
Self-rated health (1 = very bad to 5 = very good): mean (standard deviation)		3.8 (0.8)	3.4 (0.9)	<0.001
Denture usage: n (%)				<0.001
	Without dentures	1379 (53.4)	1203 (46.6)	
	With removable dentures	122 (32.0)	259 (68.0)	
	With complete dentures	36 (32.1)	76 (67.9)	
Loneliness (from 1 to 4, with higher values indicating higher loneliness levels): mean (standard deviation)		2.1 (0.6)	2.4 (0.6)	<0.001
Perceived social isolation (from 1 to 4, with higher values indicating higher perceived social isolation levels): mean (standard deviation)		1.7 (0.8)	2.1 (0.8)	<0.001
Objective social isolation (from 0 to 30, with higher values indicating higher objective social isolation levels): mean (standard deviation)		15.8 (6.3)	16.7 (5.9)	<0.001

Notes: Independent *t*-tests or Chi^2^-tests were conducted, as appropriate.

**Table 2:** Pearson correlations between key variables (total sample).

	1	2	3	4
1: Loneliness	1			
2: Perceived social isolation	0.56 ***	1		
3: Objective social isolation	0.44 ***	0.27 ***	1	
4: Oral health-related quality of life	0.23 ***	0.30 ***	0.08 ***	1
Observations	3075	3075	3075	3075

Notes: Pearson correlations are displayed. *** *p* < 0.001

**Table 3:** Pearson correlations between key variables (stratified by denture usage).

	Without Dentures				With Removable Dentures				With Complete Dentures			
	1	2	3	4	1	2	3	4	1	2	3	4
1: Loneliness	1				1				1			
2: Perceived social isolation	0.55 ***	1			0.56 ***	1			0.63 ***	1		
3: Objective social isolation	0.43 ***	0.28 ***	1		0.43 ***	0.23 ***	1		0.54 ***	0.23 *	1	
4: Oral health-related quality of life	0.24 ***	0.29 ***	0.08 ***	1	0.20 ***	0.40 ***	−0.03	1	0.28 **	0.38 ***	0.08	1
Observations	2582				381				112			

Notes: Pearson correlations are displayed. *** *p* < 0.001, ** *p* < 0.01, * *p* < 0.05.

**Table 4:** Determinants of loneliness, perceived social isolation and objective social isolation. Results of multiple linear regressions.

Independent Variables	Loneliness	Perceived Social Isolation	Objective Social Isolation
	B (robust standard errors)	B (robust standard errors)	B (robust standard errors)
Oral health-related quality of life	0.03 ***	0.06 ***	0.07 *
	(0.00)	(0.00)	(0.03)
Potential confounders	✓	✓	✓
Observations	3075	3075	3075
Adjusted R^2^	0.17	0.24	0.16
R^2^	0.18	0.24	0.17
F-value (*p*-value)	22.88 (*p* < 0.001)	37.39 (*p* < 0.001)	22.32 (*p* < 0.001)

Notes: Unstandardized beta-coefficients (B) are displayed; robust standard errors in parentheses; *** *p* < 0.001, * *p* < 0.05; Potential confounders (✓) include sex, age, marital status, education, presence of children in the same household, smoking status, alcohol intake, sports activities, vaccinated against COVID-19, presence of chronic diseases and self-rated health.

**Table 5:** Determinants of loneliness, perceived social isolation and objective social isolation (stratified by denture usage: 1. without dentures, 2. With removable dentures; 3. with complete dentures). Results of multiple linear regressions.

	Without Dentures			With Removable Dentures			With Complete Dentures		
Independent Variables	Loneliness	Perceived Social Isolation	Objective Social Isolation	Loneliness	Perceived Social Isolation	Objective Social Isolation	Loneliness	Perceived Social Isolation	Objective Social Isolation
	B (robust standard errors)	B (robust standard errors)	B (robust standard errors)	B (robust standard errors)	B (robust standard errors)	B (robust standard errors)	B (robust standard errors)	B (robust standard errors)	B (robust standard errors)
Oral health-related quality of life	0.04 ***	0.06 ***	0.08 *	0.01	0.05 ***	−0.03	0.04 *	0.06 **	0.18
	(0.00)	(0.01)	(0.04)	(0.01)	(0.01)	(0.08)	(0.02)	(0.02)	(0.17)
Potential confounders	✓	✓	✓	✓	✓	✓	✓	✓	✓
Observations	2582	2582	2582	381	381	381	112	112	112
Adjusted R^2^	0.17	0.23	0.16	0.14	0.31	0.08	0.24	0.38	0.14
R^2^	0.18	0.24	0.17	0.20	0.36	0.15	0.43	0.54	0.36
F-value (*p*-value)	19.39 (*p* < 0.001)	29.20 (*p* < 0.001)	18.51 (*p* < 0.001)	13.19 (*p* < 0.001)	58.95 (*p* < 0.001)	28.99 (*p* < 0.001)	16.09 (*p* < 0.001)	44.41 (*p* < 0.001)	22.32 (*p* < 0.001)

Notes: Unstandardized beta-coefficients are displayed; Robust standard errors in parentheses; *** *p* < 0.001, ** *p* < 0.01, * *p* < 0.05; Potential confounders (✓) include sex, age, marital status, education, presence of children in the same household, smoking status, alcohol intake, sports activities, vaccinated against COVID-19, presence of chronic diseases and self-rated health.

## Data Availability

The datasets used and analyzed during the current study are available from the corresponding author on reasonable request from all interested researchers.

## Supplementary Materials

The following are available online at https://www.mdpi.com/article/10.3390/ijerph182412886/s1, Table S1: Determinants of loneliness, perceived social isolation and objective social isolation (with dichotomized oral health-related quality of life). Results of multiple linear regressions, Table S2: Determinants of loneliness, perceived social isolation and objective social isolation (with interaction terms: denture usage x oral health-related quality of life). Results of multiple linear regressions.
