# Supplementary material for: The Association between Oral Health-Related Quality of Life, Loneliness, Perceived and Objective Social Isolation—Results of a Nationally Representative Survey
Source: Int J Environ Res Public Health. 2021 Dec 7;18(24):12886. doi: 10.3390/ijerph182412886 (PMC8701403; doi:10.3390/ijerph182412886)
Supplement: Supplementary file 1 [file ijerph-18-12886-s001.zip › ijerph-1477461-supplementary.pdf]

Supplementary Table S1. Determinants of loneliness, perceived social isolation and objective social isolation (with dichotomized oral health-related quality of life). Results of multiple linear regressions.

**Independent variables**	**Loneliness**	**Perceived social isolation**	**Objective social isolation**
Oral health-related quality of life (dichotomized: Reference category: score of zero)	0.20***	0.28***	0.46*
	(0.02)	(0.03)	(0.21)
Potential confounders	✓	✓	✓
Observations	3,075	3,075	3,075
Adjusted R^2	0.16	0.21	0.16
R^2	0.17	0.22	0.17
F-value (p-value)	21.79 (p<.001)	33.08 (p<.001)	22.21 (p<.001)

Notes: Unstandardized beta-coefficients are displayed; Robust standard errors in parentheses; *** p<0.001, ** p<0.01, * p<0.05, + p<0.10; Potential confounders include sex, age, marital status, education, presence of children in the same household, smoking status, alcohol intake, sports activities, vaccinated against Covid-19, presence of chronic diseases and self-rated health.

Supplementary Table S2. Determinants of loneliness, perceived social isolation and objective social isolation (with interaction terms: denture usage x oral health-related quality of life). Results of multiple linear regressions.

Independent variables	Loneliness	Perceived social isolation	Objective social isolation
Oral health-related quality of life	0.04***	0.06***	0.07+
	(0.00)	(0.01)	(0.04)
Denture usage (Ref.: without dentures):			
- With removable dentures	0.01	0.02	-0.21
	(0.05)	(0.05)	(0.44)
- With complete dentures	0.01	0.04	-0.28
	(0.08)	(0.05)	(0.79)
Interaction terms (Denture usage x oral health-related quality of life):			
With removable dentures (Ref.: without dentures) x oral health-related quality of life	-0.02*	-0.01	-0.01
	(0.01)	(0.51)	(0.08)
With complete dentures (Ref.: without dentures) x oral health-related quality of life	0.00	0.01	0.05
	(0.02)	(0.58)	(0.14)
Potential confounders	✓	✓	✓
Observations	3,075	3,075	3,075
Adjusted R^2	0.17	0.24	0.17
R^2	0.18	0.23	0.16
F-value (p-value)	20.49 (p<.001)	32.87 (p<.001)	19.71 (p<.001)

Notes: Unstandardized beta-coefficients are displayed; Robust standard errors in parentheses; *** p<0.001, ** p<0.01, * p<0.05, + p<0.10; Potential confounders include sex, age, marital status, education, presence of children in the same household, smoking status, alcohol intake, sports activities, vaccinated against Covid-19, presence of chronic diseases and self-rated health.
